# Crosstalk reduction of integrated optical waveguides with nonuniform subwavelength silicon strips

**DOI:** 10.1038/s41598-020-61149-1

**Published:** 2020-03-11

**Authors:** Yang Yang, Yinghui Guo, Yijia Huang, Mingbo Pu, Yanqin Wang, Xiaoliang Ma, Xiong Li, Xiangang Luo

**Affiliations:** 1 State Key Laboratory of Optical Technologies on Nano-Fabrication and Micro-Engineering, Institute of Optics and Electronics, Chinese Academy of Sciences, P.O. Box 350, Chengdu, 610209 China; 2 School of Optoelectronics, University of Chinese Academy of Sciences, Beijing, 100049 China

**Keywords:** Nanophotonics and plasmonics, Silicon photonics

## Abstract

Suppression of the crosstalk between adjacent waveguides is important yet challenging in the development of compact and dense photonic integrated circuits (PICs). During the past few years, a few of excellent approaches have been proposed to achieve this goal. Here, we propose a novel strategy by introducing nonuniform subwavelength strips between adjacent waveguides. In order to determine the widths and positions of nonuniform subwavelength strips, the particle swarm optimization (PSO) algorithm is utilized. Numerical results demonstrate that the coupling length between adjacent waveguides is increased by three (five) orders of magnitude in comparison with the case of uniform (no) subwavelength strips. Our method greatly reduces crosstalk and is expected to achieve a highly compact integrated density of PICs.

## Introduction

Over the past decades, much attention has been paid on the development of silicon photonics because of its compatibility with complementary metal oxide semiconductor (CMOS) technology^1,2^. Along with its further development, more and more waveguide elements need to be integrated on the silicon-on-insulator (SOI) platform^3^. Though various optical elements have been studied so far, such as transceivers, couplers/inverse taper, subwavelength gratings, multiplexers and so on^4–9^, the packing density of photonic integrated circuits (PICs) is still not as high as their electronic counterparts^10^. The reason is that the diffraction limit hinders the further shrinking of waveguide^11^.

During the past few years, great efforts have been paid to improve the integration density of PICs. One intuitive method is the use of plasmonic waveguides or metal-dielectric hybrid structures that can overcome the diffraction limit to further shrink the device footprint^12–14^. However, the large ohmic losses in metal limit its application in PICs. Alternatively, if we can effectively reduce the crosstalk between adjacent waveguide components, the minimum spacing will be greatly decreased and the packing density increased correspondingly. Recently, waveguide super-lattices^15,16^ and nanophotonic cloaking^17^ have been proposed to reduce crosstalk. The fundamental results show that negligible crosstalk can be obtained at telecommunication wavelengths.

Recently, subwavelength silicon gratings have been brought into optical waveguide and on-chip architectures to provide new degrees of freedom to control the guided wave in PICs^18–21^. As a consequence, the past few years have witnessed the emergence of a couple of compact waveguide devices including polarization/wavelength splitter^22^, polarization/mode converters/rotators^23,24^ and ultra-sharp waveguide bends^25^, integrated photonic switches^26^. Besides harnessing the guided wave, recent theoretical investigations show that subwavelength silicon gratings can be also utilized for evanescent wave manipulation^27^. Interestingly, the anisotropic gratings strips possess low effective index in direction normal to the gratings for total internal reflection in the core waveguide. Simultaneously, the anisotropic silicon gratings exhibit high index in the direction parallel to the interface for rapid decay of evanescent waves leaked from the core waveguide. Following this principle, Amin Khavasi *et al*. introduced two subwavelength strips between two neighboring waveguides^18^. Experimental results indicated that all-dielectric metamaterials can achieve a highly constrained mode and the coupling length is increased by ten times compared to the case without subwavelength strips. Yu *et al*. subsequently carried comprehensive studies regarding the effect of different geometries and their physical dimensions on the performance of crosstalk reduction^28^. With the same waveguide sizes, they increased the coupling length by two orders of magnitude when more than three silicon strips are placed between two adjacent waveguides.

It should be noted that all of the aforementioned works are based on uniform subwavelength gratings/strips, which can be taken as a homogenous material in the macroscopic picture. It is intuitive to know that a nonuniform structure possesses more design freedom than a uniform counterpart^29^. Bian considered uniform strips and the obvious question, inspired by other work in photonic optimization^30–38^, a computationally-optimized nonuniform design could be even better.

## Results

**Figure 1:**
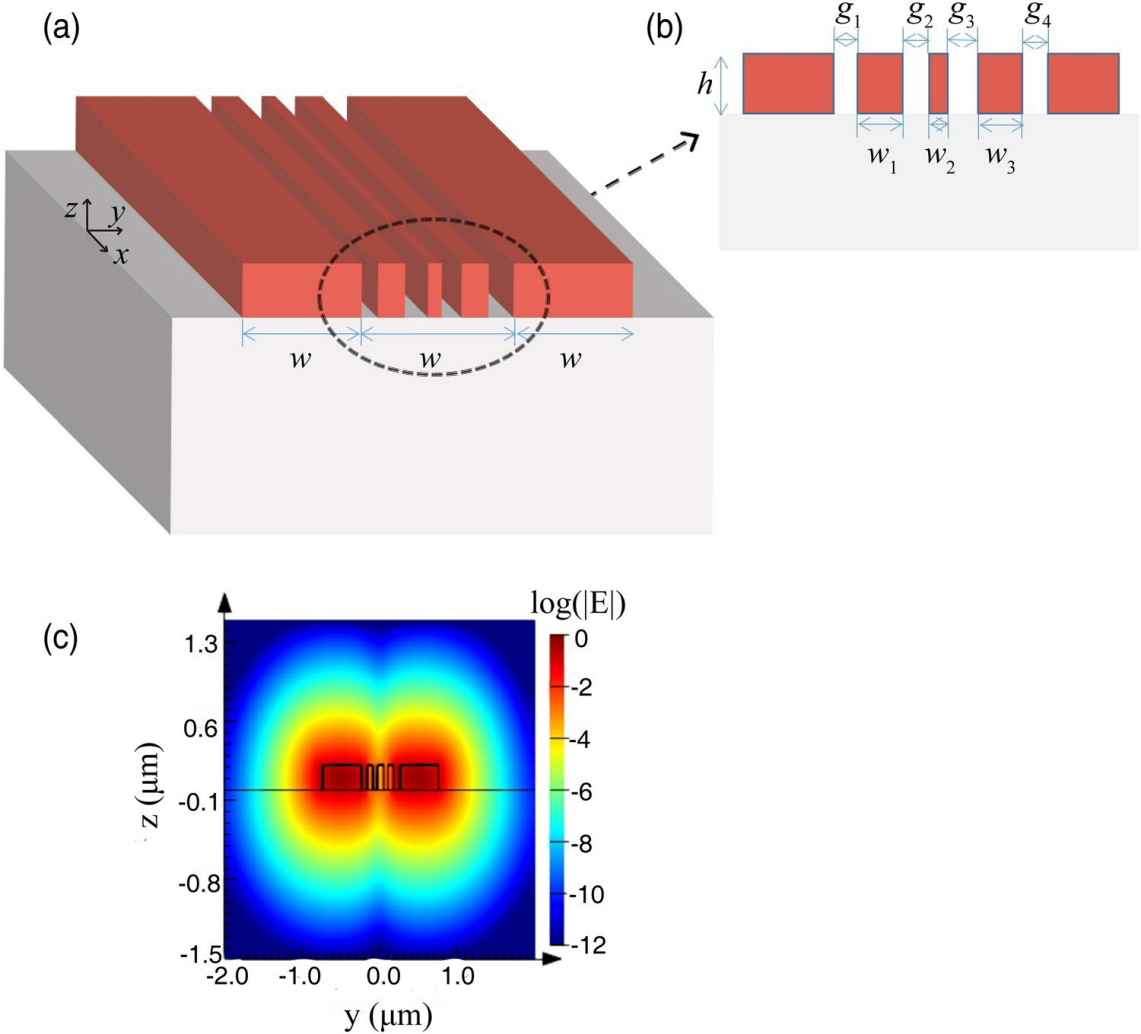
Simulation model and mode field distribution. (**a**) The proposed configuration that comprises a nonuniform silicon strip array inserted between the silicon waveguide pair. The substrate and cladding are silica and air, respectively. The width and height of two identical silicon ridge waveguides are 500 nm and 220 nm, respectively. (**b**) An enlarged view of the structure. The widths of three strips are *w*_*1*_*, w*_*2*_*, w*_3_, respectively. The widths of four gaps are *g*_1_*, g*_2_*, g*_3_*, g*_4_ respectively. (**c**) The electric field distribution of the waveguide cross section is plotted in log scale.

Figure 1(a,b) show the proposed configuration that incorporates several subwavelength silicon strips lying in-between two adjacent single-mode SOI waveguides. The width, height, and edge to edge separation of the core waveguides are set to be 500 nm, 220 nm and 500 nm, respectively. Since the separation between single-mode SOI waveguides is only 500 nm (~λ/3), strong evanescent waves coupling between them will occur, accompanied by strong crosstalk and catenary shaped field^39^ (a hyperbolic consine function). Therefore, the motivation of this paper is to effectively suppress crosstalk by introducing nonuniform subwavelength structures (three subwavelength strips are utilized here as a representative example) between two neighboring waveguides. Since strong evanescent waves coupling or crosstalk will result in a short coupling distance, a useful measure of crosstalk strength is characterized by the coupling distance, which can be taken as the figure of merit (FOM) of the optimization design.

Intrinsically, the crosstalk is a consequence of the interaction between the modes in two adjacent waveguides. In this circumstance, the coupling between the original mode in each guide gives rise to two new supermodes that are simultaneously supported by two strong coupled waveguides, i.e., one symmetric (even) supermode and one antisymmetric (odd) supermode with the propagation constants denoted as *β*_+_ and *β*_*−*_, respectively. The coupling length *L*_c_ is defined as the distance over which a *π* phase difference accumulates between the two modes^40^, and thus can be expressed as:

1 $${L}_{c}=\frac{\pi }{2\Delta \beta }=\frac{\pi }{{\beta }_{+}-{\beta }_{-}}$$

Substituting the relationship between propagation constants is related to the mode effective index i.e., *β*_+_  = *2πn*_*e*_*/λ* and *β*_−_ = *2πn*_*o*_*/λ* into Eq. (1), we can arrive at:

2 $${L}_{c}=\frac{\lambda }{2({n}_{e}-{n}_{0})}$$

where *n*_*e*_ and *n*_*o*_ are the real parts of the mode indexes for the even and odd modes, respectively (see Supplementary Fig. S1 for details). According to Eq. (2), when the mode index difference between them is infinitely small, the coupling distance will become infinitely large. Therefore, the key of the design is finding proper nonuniform subwavelength structures geometries to make the mode index difference as small as possible. Although one can obtain a rough estimate of the mode refractive index based on effective material theory when the waveguide dimensions are larger than the wavelength of light, it is not practical when the waveguide dimensions fall in the subwavelength scale^41^. The proposed silicon waveguide structures were investigated numerically by solving Maxwell’s equations on a cross-sectional mesh of the waveguide using the eigenmode solver of the finite difference eigenmode method (FDE) based software MODE solutions. The core of the FDE method is that Maxwell’s equations are formulated into a matrix eigenvalue problem after meshing the waveguide geometry by using the finite difference algorithm, and then, to obtain the effective index and mode profiles of the waveguide modes by using sparse matrix techniques (Lumerical Inc.)^42^.

**Figure 2:**
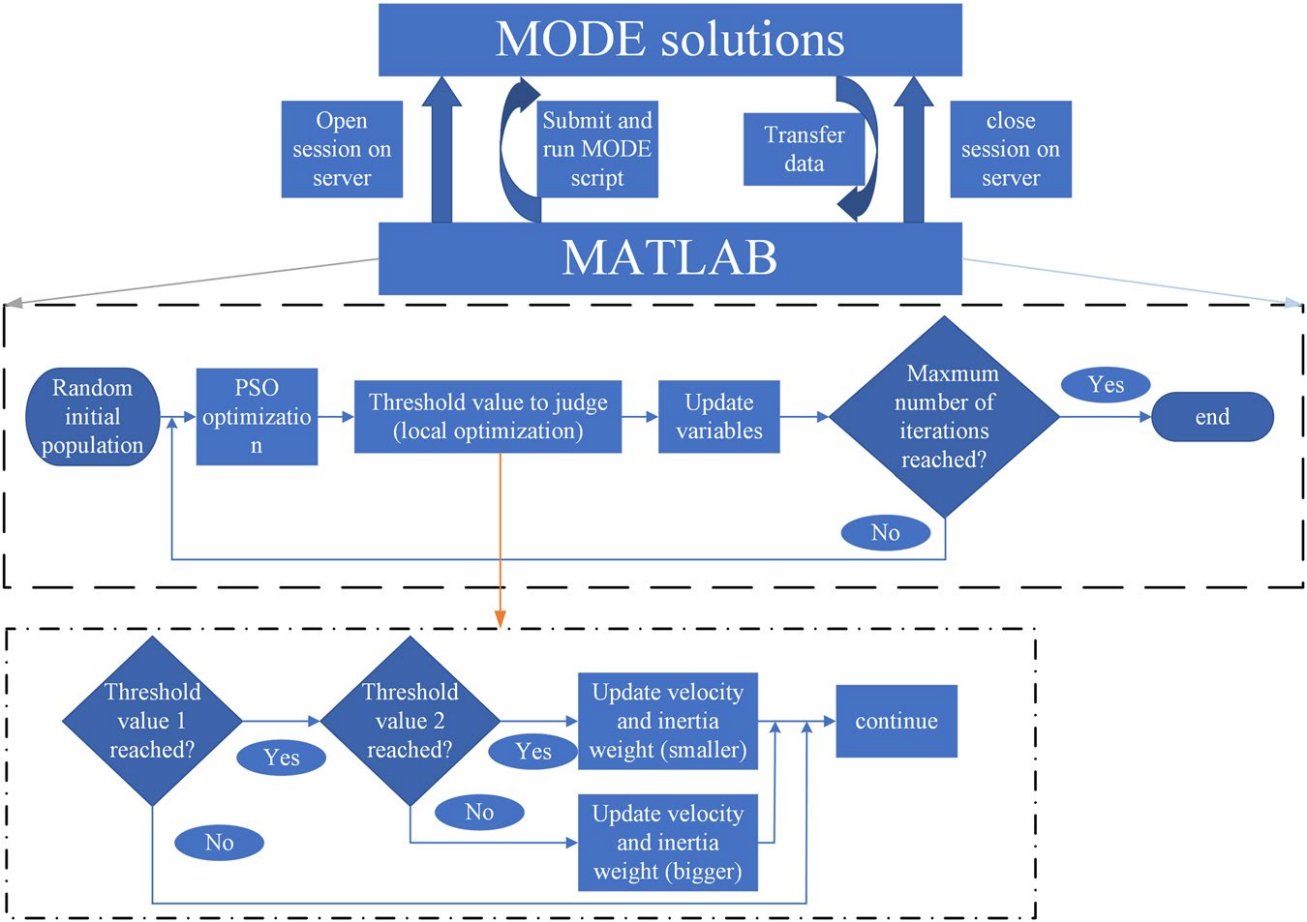
The flow chart of scripted MODE solutions with matlab.

Since the mode index is highly sensitive to the geometries, separation distance, and ambient conditions, the introduction of several nonuniform subwavelength structures between the two coupled waveguides gives rise to a nontrivial design space for decreasing the crosstalk. Since it is inefficient to find an excellent design by full parameters sweeping, we alternatively resort to optimization algorithms in our design. Among various optimization algorithms, particle swarm optimization (PSO) has been widely used in the optimization of a myriad of optical waveguide devices, e.g., waveguide crossing^43^, optical phased arrays^44^, compact silicon grating coupler^45^. Initially, several simulations with different coefficients were performed using the standard PSO (SPSO) setup, and the results were always fluid. The desired coupling length was mainly varying between 10^7^~10^8^ μm and sometimes greater than 10^8^ μm which meant that the program might be stuck in the local optimum. In order to tackle this problem, the computationally-optimized method is adopted here. Fortunately, the commercial software MODE Solutions has provided an application programming interface (API) to the MATLAB so that we can run MODE Solutions with matlab. Among them, the MODE Solutions provides a comprehensive optical waveguide design environment, in which FDE solvers could be used to calculate the effective index precisely. The improved PSO algorithm for avoiding local optimization is implemented by MATLAB software. A flow diagram of the simulation for the optimization process is shown in Fig. 2.

The simulation includes two significant processes: one is the data exchange between MATLAB and MODE, and another is the iterative operation of optimization algorithm in MATLAB. Firstly, a set of widths and gaps are randomly generated in MATLAB within the specified range, which is transferred to MODE by using API to generate subwavelength structure models with different widths and separations. The mode effective index which is calculated by using the FDE solver is passed back to MATLAB for calculating coupling length. The maximum coupling length and the width information of its corresponding subwavelength structure are stored. Next, we move on to the second part. The population of each iteration consists of 30 particles. The currently obtained coupling length, widths and gaps generated in each iteration step are compared with the previous best results, and the maximum coupling length as well as corresponding widths and gaps are updated each time. The performance of SPSO is affected by changing the values of its coefficients. Inspired by an adaptive approach^46^, a dynamic maximum flight speed and an inertia weight are chosen by selecting the appropriate range. The inertia weight provides a restriction in position jump: low values allow particles to roam around the target positions, while high values provide a large movement orientated to target. Maximum flight speed determines how far the particles in PSO can fly. Initially, the inertia weight and maximum flight speed are set to be 0.6 and 0.05, respectively. To a certain extent, keeping the dynamic change of speed within the specified range can avoid falling into local optimization. Dynamic flight speed is applied to update the position. Here, we set two sets of the inertia weight (0.55~0.65 under the threshold 1 and 0.65~0.75 under the threshold 2) and flight speed (0.005~0.01 under the threshold 1 and 0.01~0.05 under the threshold 2) according to the requirements of two different thresholds. In the initial attempt, we find that it is difficult to make a change once the coupling length exceeds 10 m. Therefore, in order to avoid falling into local optimization, we use the “jump long distance, search small distance” approach to improve the PSO algorithm. And two threshold values (threshold 1 is 10^7^ μm and threshold 2 is 10^8^ μm) are set in advance for judgment. Specifically, when the coupling length reaches the threshold value 1 and maintains no change for a certain amount of generations, a slightly larger inertia weight and a flight speed (inertia weight: 0.65~0.75, flight speed: 0.01~0.05) are utilized to endow a new position to the parameters of swarms, and subsequently, a small range of iterative search (inertia weight: 0.55~0.65, flight speed: 0.005~0.01) is performed. If the results remain the same after a certain number of iterations (such as, ten generations), a large range of jumps is applied again and the circle continues until the coupling length exceeds threshold value 2 or the maximum number of iterations is reached.

In order to verify our proposal, different aspects are presented in the following. The first design is a pair of Si ridge waveguides with an edge-to-edge separation of 500 nm, with three subwavelength strips in between to reduce crosstalk. The second design is similar to the first one but with an edge-to-edge separation of 450 nm. Meanwhile, the electric field intensity distributions of both cases are plotted so as to see the coupling process in a more intuitive way.

**Figure 3:**
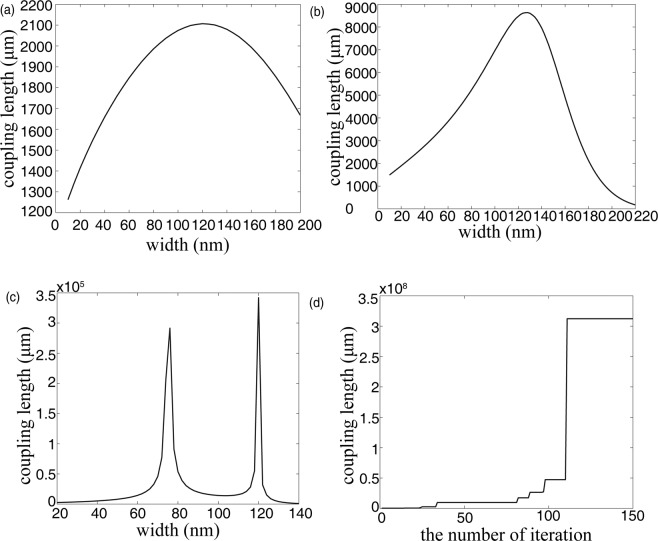
Optimization process and results. Effect of the strip width on the coupling length for uniform configurations with (**a**) one strip, (**b**) two strips and (**c**) three strips. (**d**) The iterative result obtained by using PSO algorithm with three nonuniform strips.

Uniform subwavelength strips (e.g. one strip, two and three strips with the same width and separation) are first investigated to compare with the results of the ref. ^28^. The results shown in Fig. 3(a–c) indicate that a good agreement between them is found and the small discrepancy is caused by different software, demonstrating our convergence test is justification (see Supplementary Table S1). Next, we take three strips as example to explore the influence of the introduction of subwavelength structures on suppressing crosstalk. Figure 3(d) shows the FOM varies with the number of iterations, where the coupling length is calculated according to the theoretical formula shown in Eq. (2), with the effective indexes obtained by using the FDE solver. After more than 100 iterations, the coupling length reaches 312.2 m, three orders of magnitude improvement than the result obtained in the ref. ^28^. The results demonstrated that the proposed strategy of introducing nonuniform subwavelength strips can be used as a promising candidate in suppressing crosstalk between two neighboring waveguides. The optimization widths of gaps and strips (*g*_1_*, w*_1_*, g*_2_*, w*_2_*, g*_3_*, w*_3_*, g*_4_) are 77 nm, 72 nm, 58 nm, 86 nm, 58 nm, 72 nm, 77 nm, respectively. It should be noted that the optimization design can be fabricated by advanced 193 nm immersion lithography technology with an achieved feature size down to 50 nm^47^. The analysis of the fabrication imperfection of our proposed structures and its sensitivity to wavelength are also studied (see Supplementary Fig. S2).

**Figure 4:**
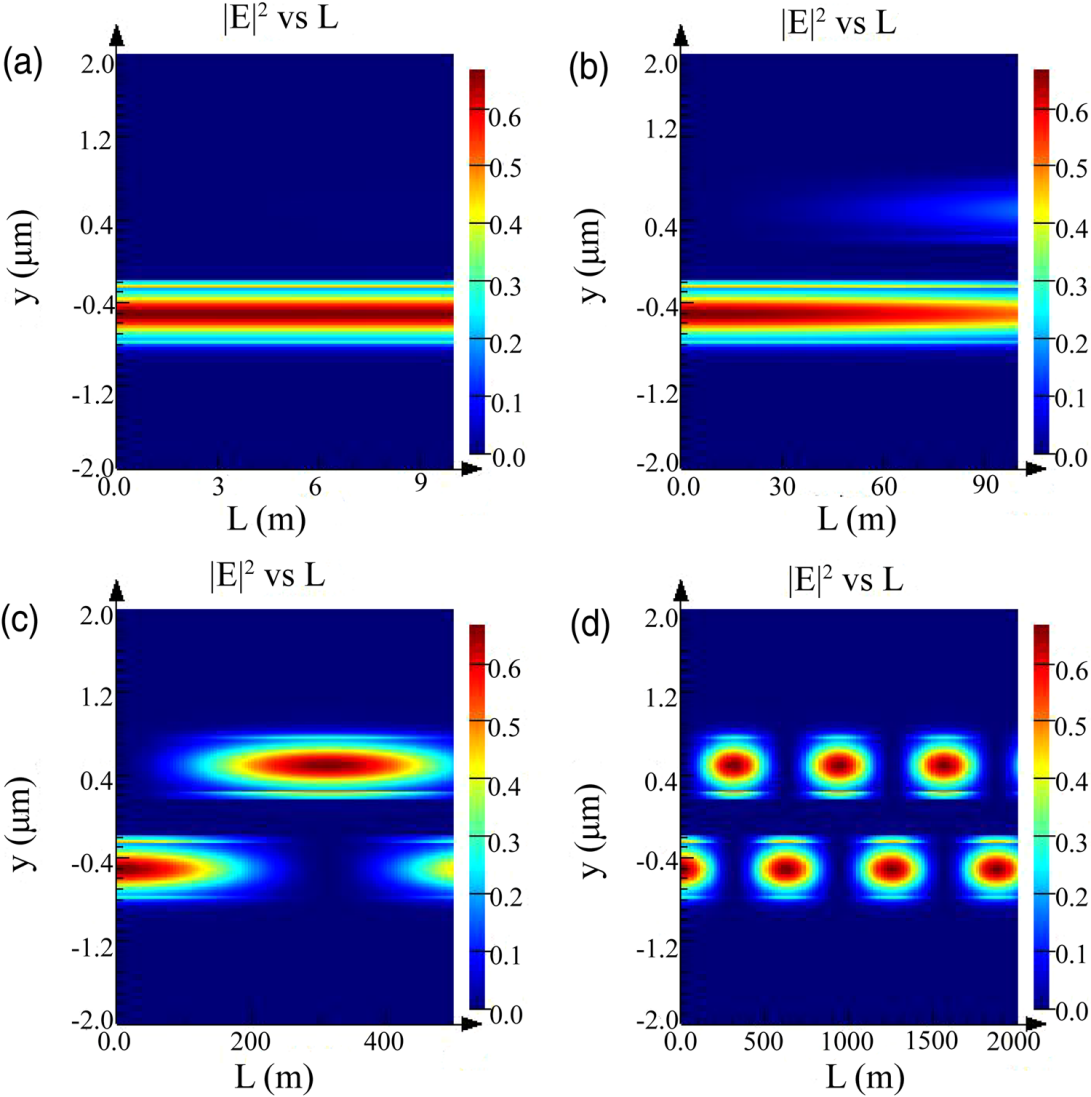
The electric field intensity distribution versus y and L. (**a**) Electric field intensity distribution with a propagation length of 10 m. No coupling occurs between two waveguides. (**b**) Electric field intensity distribution with a propagation length of 100 m, where coupling is going to happen. (**c**) Electric field intensity distribution with a propagation length of 500 m. It could be seen that a complete coupling process is generated between two waveguides. (**d**) Electric field intensity distribution with propagation length of 2000 m.

In order to study the influence of the introduction of nonuniform subwavelength strips on the crosstalk between waveguides in Fig. 1, the electric field intensity distribution along the propagation direction was examined. The acquisition of electric field intensity distribution is divided into two steps. The first step is to calculate the field distribution along the propagation direction for both supermodes (even mode and odd mode) through a “propagate” command packaged by the software or FDE solver, and the second step is to accumulate these field distributions to obtain the total propagation field. The concrete implementation is achieved through scripts. It can be seen from Fig. 4(a,b) that the fundamental mode can be transmitted forward for more than 100 m without crosstalk occurring, which is superior to the result previously reported^28^. The coupling length obtained from Fig. 4(c) agrees well with the result we get in Fig. 3(d). Multiple coupling processes occur when the propagation length is far enough, as shown in Fig. 4(d).

**Figure 5:**
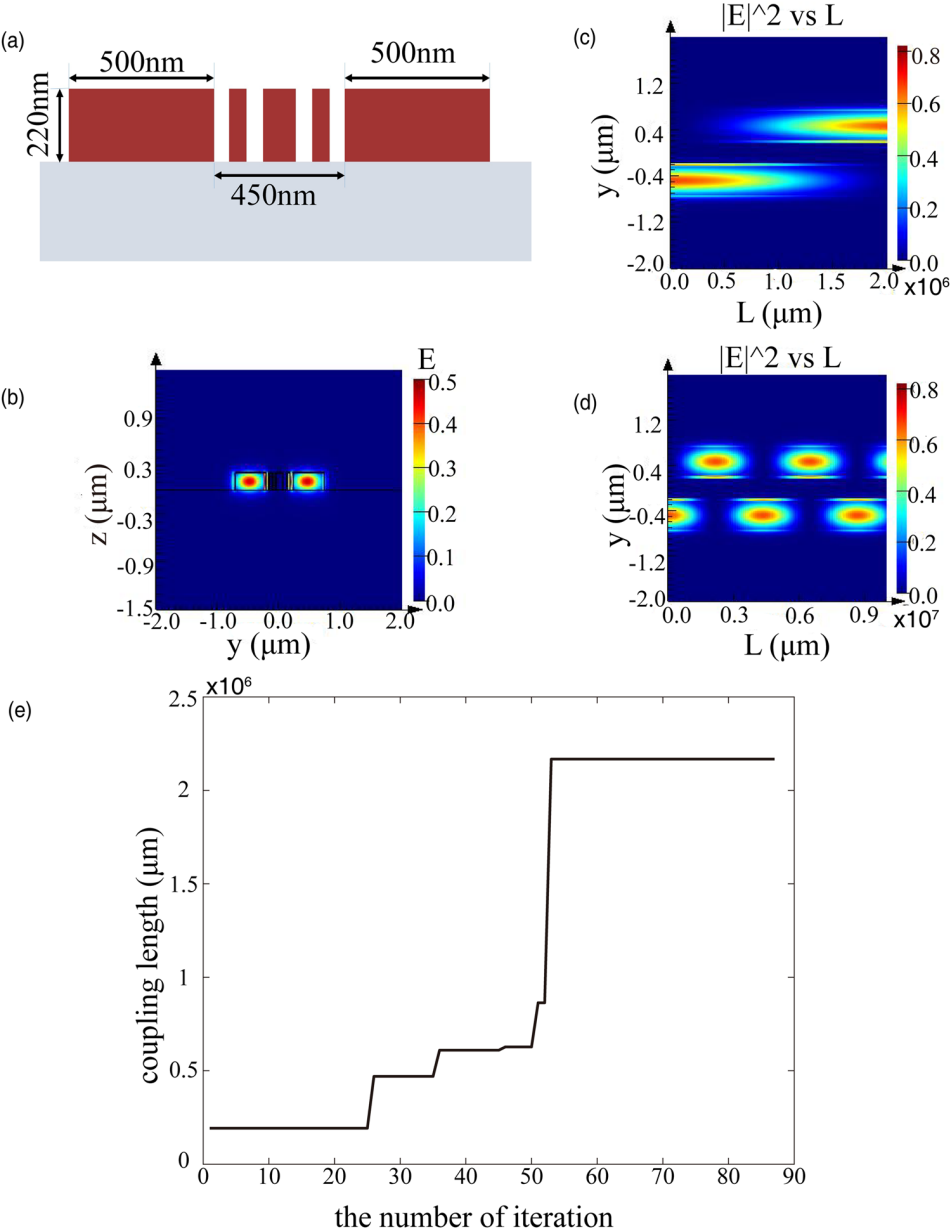
Simulation results of evanescent coupling waveguides with a separation gap of 450 nm. (**a**) Schematic diagram of structure with a separation gap of 450 nm. (**b**) Mode intensity distribution in waveguide cross section. (**c**,**d**) Distribution of light fields with different propagation lengths. (**e**) The optimization progress for evanescent coupling waveguide with a separation gap of 450 nm.

Further research shows that the proposed crosstalk suppressing method can work well for smaller waveguide spacing, as shown in Fig. 5(a). We give a schematic that two silicon ridge waveguides are separated from each other with an edge to edge distance of 450 nm, achieving the coupling length of 2.167 m. Figure 5(b) indicates that strong confinement occurs in the ridge waveguide. The simulation result can be obtained from Fig. 5(c,d), which is close to the theory result in Fig. 5(e). Different from the previous setting, we set the inner mesh size to be 500 nm, i.e. an expansion of 25 nm on each side. The optimization widths of gaps and strips (*g*_1_*, w*_1_*, g*_2_*, w*_2_*, g*_3_*, w*_3_*, g*_4_) are 52 nm, 60 nm, 57 nm, 112 nm, 57 nm, 60 nm, 52 nm, respectively. Furthermore, smaller waveguide spacings are tested. (See Supplementary Fig. S3 for detail).

Even if the edge to edge separation of waveguides is set to be 300 nm, the coupling length can still reach 1000 μm. Although the coupling distance can be further improved by adopting better optimized algorithm and more complex structures with stronger anisotropy, the increase in coupling length seems to have approached to an upper limit owing to the limitations of available materials and fabrication resolution. Furthermore, evanescent waves cannot be completely eliminated, even if they decay sharply between waveguides, according to the theory of Jahani *et al*. This means that crosstalk must occur between two parallel waveguides, indicating that the coupling length is also limited, but may not be the value we found. As the figure shown above, as the distance between two waveguides becomes smaller, the coupling length decreases. Though unavoidably, our method can still promote the development of silicon photonics in terms of integration and miniaturization due to the effect of great crosstalk suppression.

## Discussions

In this paper, we introduce nonuniform subwavelength strip arrays between integrated optical waveguides. Compared with a pair of waveguides with introducing uniform strip arrays, the coupling length is improved by three orders of magnitude, and it is five orders of magnitude greater than the case without strip arrays. It is shown that crosstalk is also suppressed well for waveguides with even smaller spacing, e.g., 450 nm, 400 nm, 350 nm and 300 nm. Though we have to compromise the coupling length and spacing, further reduction in spacing with good coupling length is expected in the future. The introduction of nonuniform strips is expected to realize more intensive photonic integration platform. The reduction of crosstalk is beneficial for the further development of on-chip spectrometer^48^, automatic cars^49^, and other applications^50^.

## Supplementary information

Supplementary information.
