# Supplementary material for: Crosstalk reduction of integrated optical waveguides with nonuniform subwavelength silicon strips
Source: Sci Rep. 2020 Mar 11;10:4491. doi: 10.1038/s41598-020-61149-1 (PMC7066159; doi:10.1038/s41598-020-61149-1)
Supplement: Supplementary file 1 — Supplementary information. [file 41598_2020_61149_MOESM1_ESM.docx]

**Supplementary Information for:**

**Crosstalk reduction of integrated optical waveguides with nonuniform subwavelength silicon strips**

Yang Yang,^1, 2*^ Yinghui Guo,^1, 2*^ Yijia Huang,^1, 2^ Mingbo Pu,^1, 2^ Yanqin Wang,^1, 2^ Xiaoliang Ma,^1, 2^ Xiong Li,^1, 2^ and Xiangang Luo^1, 2*^

^1^State Key Laboratory of Optical Technologies on Nano-Fabrication and Micro-Engineering, Institute of Optics and Electronics, Chinese Academy of Sciences, P.O. Box 350, Chengdu 610209, China

^2^School of Optoelectronics, University of Chinese Academy of Sciences, Beijing 100049, China

^*^These authors contribute equally to this work

Correspondence and requests for materials should be addressed to X.G.L. [(lxg@ioe.ac.cn)](mailto:(lxg@ioe.ac.cn))

S1. Supermodes between two parallel waveguides introducing subwavelength structures.


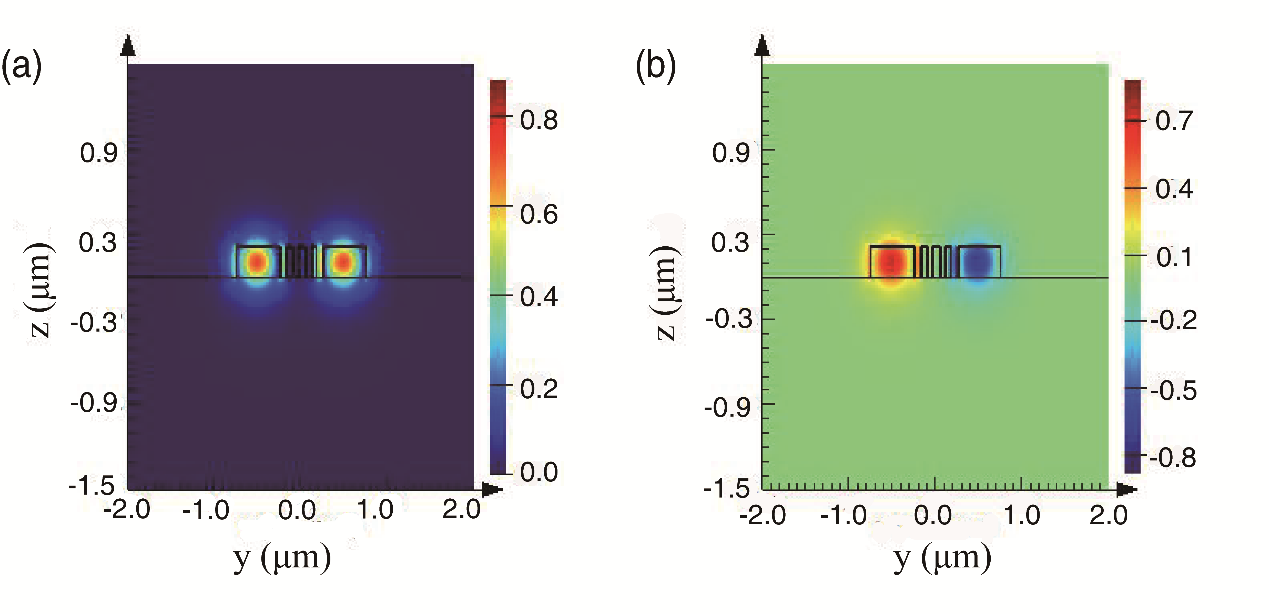


**Supplementary Figure 1.** Mode field distribution between two parallel waveguides introducing three strips. (a) Symmetrical mode. (b) Asymmetrical mode.

S2. Analysis of mesh accuracy

Due to the subwavelength characteristic size of waveguide strips, high simulation resolution is required to ensure the accuracy of simulation results. However, the improvement of simulation accuracy will in turn leads to the increase of simulation time. As shown in Supplementary Table S1 (case 1), simulation time exceeds 10 min when the entire simulation space is covered with mesh accuracy of 1 nm. On the other hand, a bigger simulation accuracy generally shortens the simulation time but may also result in the truncation of the energy and thus decreases the simulation accuracy. As shown in Supplementary Table S1 (case 3-9), the reduction of simulation time results in a huge error, that is, the difference of effective index Δn_eff_ (n_e_-n_o_) fluctuate greatly. Therefore, it is necessary to do a convergence test before the simulation to determine the appropriate simulation area and mesh resolution that would make a good balance between simulation accuracy and simulation time. Convergence test was implemented in the modeling process, where the silicon structures and the silica substrate use the refractive index data from Palik and the refractive index of the air cladding is 1. The simulation area is determined as to where the mode strength decays to less than -10dB compared to the maximum value. Finally, the simulation cross section is determined to be 4 µm long and 3 µm wide, and mesh cells of 50 in two directions are respectively collected in the whole simulation area.

As shown in Supplementary Table S1, different simulation areas and mesh accuracy are tested. In order to make a good tradeoff between the simulation accuracy and time, two more different mesh settings are utilized to cover the whole simulation area. The utmost high mesh resolutions of dy = 1 nm, dz = 1 nm are applied at the inner area (0.56 μm long and 0.22 μm wide), where the electric field changes abruptly around the two core waveguides and the subwavelength gradient strips. High mesh resolutions of dy = 4 nm, dz = 1 nm are applied at the middle transmission area (2 μm long and 2 μm wide) between the inner area and outmost area. The setting of mesh override regions ensures the accuracy of simulation results and lays a foundation for the next application of co-simulation. Limited by the hardware resources we use, it seems to be difficult to use a smaller mesh accuracy (less than 1 nm). Therefore, better computer configurations need to be applied in order to further improve the accuracy of simulation results.

**Supplementary Table S1.** The difference of effective index with different mesh accuracy.

| [77,72,58,86,58,72,77] units: nm | | | | | | |
| --- | --- | --- | --- | --- | --- | --- |
|  | Mesh area (y, z)  Units: um | | Mesh accuracy (dy, dz) units: nm | | Δn_eff_  ×10^-12^ | Time |
|  | middle | inner | middle | inner |  |  |
| 1 | (4,3) |  | (1,1) |  | 3071 | >10min |
| 2 | (2,2) |  | (1,1) |  | 2698 | 4min |
| 3 | (2,2) |  | (2,1) |  | 419339 | 1min40s |
| 4 | (2,2) |  | (4,1) |  | 519754 | 1min |
| 5 | (2,2) |  | (5,1) |  | 468157 | 48s |
| 6 | (2,2) |  | (1,2) |  | 123395 | 2min |
| 7 | (2,2) |  | (2,2) |  | 617969 | 56s |
| 8 | (2,2) |  | (4,2) |  | 1124337 | 30s |
| 9 | (2,2) |  | (1,4) |  | 431177 | 1min |
| 10 | (2,2) | (0.56,0.22) | (5,1) | (1,1) | 8922 | 1min42s |
| 11 | (2,2) | (0.56,0.22) | (4,1) | (1,1) | 2482 | 1min52s |
| 12 | (2,2) | (0.56,0.22) | (2,1) | (1,1) | 4154 | 2min40s |

S3. Fabrication imperfections of the structure and its sensitivity to wavelength

In the actual manufacturing process, accurate control of the width of the subwavelength structures is a challenging work. So, we need to ensure that our structure still performs well within a reasonable margin of error. As shown in Supplementary Fig. 2(a), we measure the sensitivity of the structure to the wavelength in the range of ±50 nm. The coupling length can still be improved by 1~6 orders of magnitude compared with the pure waveguides without introducing subwavelength structures. In addition, the error analysis of the structure is conducted at ±10 nm, as shown in Supplementary Fig. 2(b). At least an order of magnitude improvement is still in effect. These results further validate the robustness of our method to crosstalk suppression with realistic fabrication imperfections.


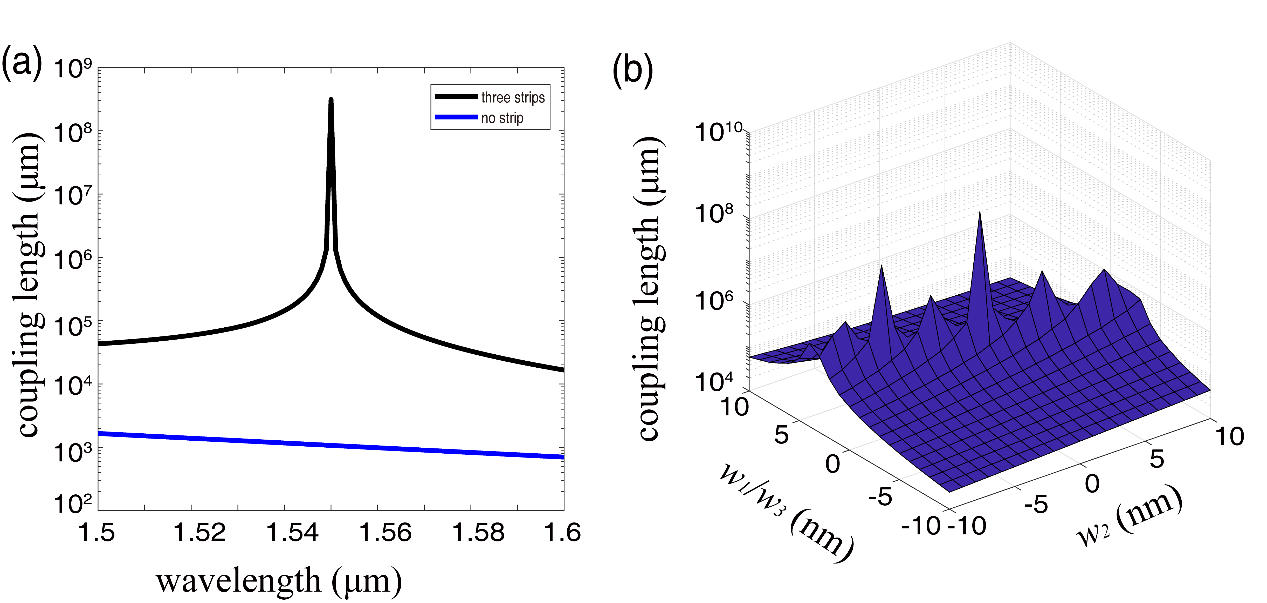


**Supplementary Figure 2.** (a) Sensitivity of the coupling length to wavelength for configurations with and without three strips. Variations in the wavelength of +/-50 nm are considered in this study. (b) Dependence of the coupling length on the strip widths with three strips. Variations in strip widths of +/-10 nm are considered in this study.

S4. The iterations for different waveguide spacings

**
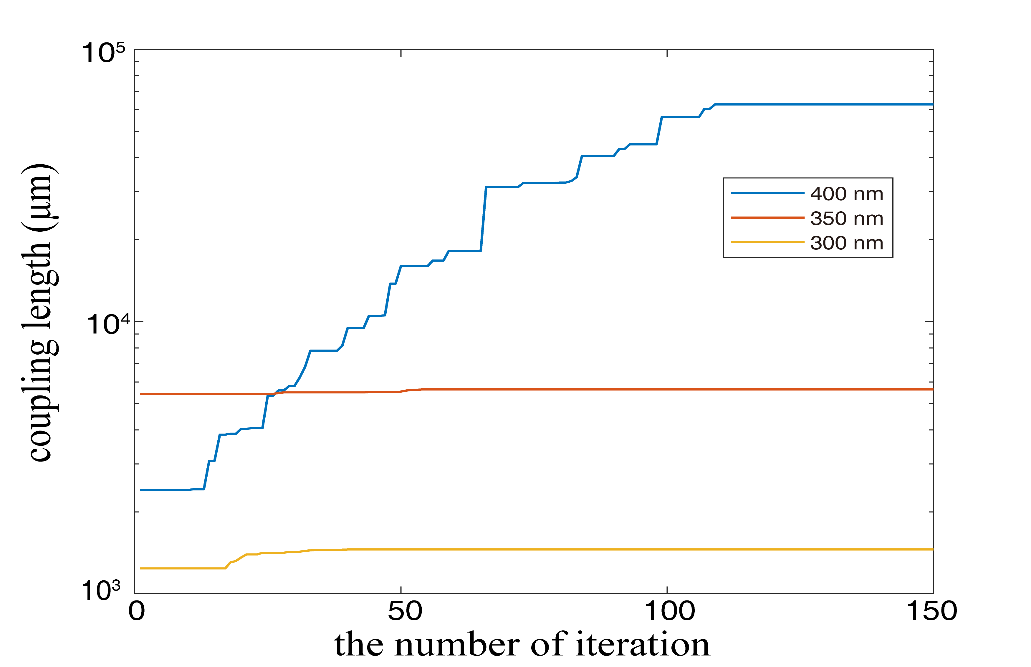
**

**Supplementary Figure 3.** The iterations at different waveguide intervals with an edge-to-edge separation of 400 nm, 350 nm and 300 nm, respectively.
